# Syndromic anorectal malformation associated with Holt–Oram syndrome, microcephaly, and bilateral corneal opacity: a case report

**DOI:** 10.1186/s13256-016-1011-7

**Published:** 2016-08-05

**Authors:** Usang E. Usang, Thomas U. Agan, Akan W. Inyang, John-Daniel C. Emehute, Itam H. Itam

**Affiliations:** 1 Division of Paediatric Surgery, Department of Surgery, University of Calabar/University of Calabar Teaching Hospital (UCTH), Calabar, Cross River State Nigeria; 2 Department of Obstetrics and Gynaecology, University of Calabar/University of Calabar Teaching Hospital (UCTH), Calabar, Cross River State Nigeria

**Keywords:** Syndromic, Ano-rectal malformation, Holt–Oram syndrome, Microcephaly, Corneal opacity

## Abstract

**Background:**

The occurrence of an anorectal malformation with Holt–Oram syndrome, microcephaly, and bilateral corneal opacity is rare and to the best of our knowledge has not previously been reported in the literature. Hence, there is a need to document our experience in this case and learn as much as possible from it.

**Case presentation:**

We present the case of a Nigerian female neonate with a postnatal diagnosis of syndromic anorectal malformation associated with Holt–Oram syndrome, microcephaly, and bilateral corneal opacity. The infant had successful staged correction of her anorectal malformation but developed a metastatic Wilms’ tumor and died before other corrective procedures could be instituted.

**Conclusions:**

An anorectal malformation is here reported to occur with Holt–Oram syndrome, an association that has not been reported previously. To enhance the prognosis and quality of life of children with syndromic anorectal malformation, prenatal ultrasound monitoring of high-risk pregnancies and expertise in prenatal detection of congenital anomalies are invaluable in antenatal care.

## Background

Anorectal malformations (ARMs) are rare congenital birth defects that occur in approximately 1 in 1500 to 1 in 5000 live births [1]. Associated congenital anomalies occur in 43–71 % of children with ARMs [2]. In up to 50 % of cases, the associated birth defects are thought to be syndromic [3]. The overall prognosis and quality of life of children with ARMs depend to a great extent on the presence and severity of these associated anomalies [4]. Therefore, a high index of suspicion and meticulous evaluation of children with ARMs are essential so as to promptly detect the coexisting anomalies often responsible for high morbidity and mortality [5].

We recently managed a Nigerian female infant with syndromic ARM associated with Holt–Oram syndrome (HOS) in conjunction with microcephaly and bilateral corneal opacity. This combination of anomalies with ARM has not been previously reported and so needed to be carefully documented.

## Case presentation

A 4-week-old female infant was born to a thirty eight-year-old para eight plus three with four alive Nigerian mother of the Ekoi tribe in Cross River State through non-consanguineous parentage. From birth, she presented with microcephaly, bilateral microphthalmia, bilateral corneal opacity, bilateral radial club-hand, congenital absence of both thumbs, and an absent anus. The pregnancy was uneventful and was carried to term under supervised antenatal care (ANC) that was booked at 12 weeks’ gestation in a private health facility. Two prenatal ultrasound (US) scans at 5 and 8 months’ gestation did not diagnose any anomalies.

The infant’s mother had no history of ingestion of herbal or non-prescription medications and no intake of alcohol or tobacco in any form throughout the duration of the pregnancy. She had no exposure to irradiation, no viral infections, and no rashes during the pregnancy. However, she did have a history of loss of three previous babies who died from unrelated causes.

Due to premature contractions and malaria in the second trimester, the mother was treated with nitrazepam, salbutamol, Calcium Sandoz (calcium lactate gluconate and calcium carbonate combination), and a dihydroartemisinin/piperaquine phosphate combination. Delivery was completed vaginally and the infant cried after several stimulations.

A clinical examination revealed a full-term female infant with a birth weight of 2.5 kg. She was 44 cm long and had an occipitofrontal circumference of 29 cm. Her heart rate was 140 beats per minute and a pansystolic murmur was heard in heart sounds I and II, which was diagnosed as a ventricular septal defect (VSD) on echocardiography.

**Fig. 1:**
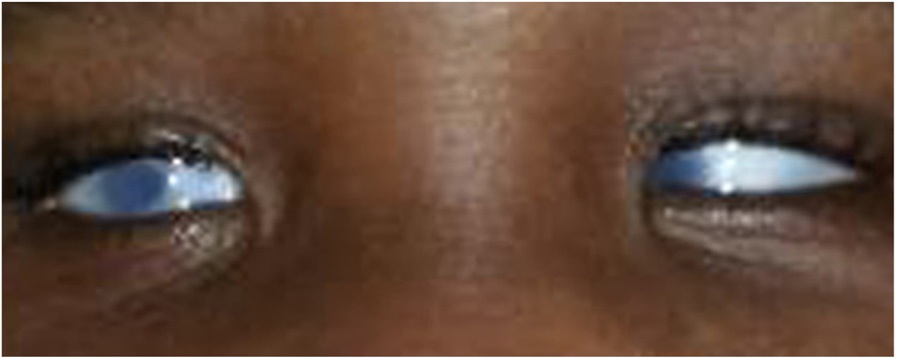
Bilateral corneal opacity: picture at 15 months of life

**Fig. 2:**
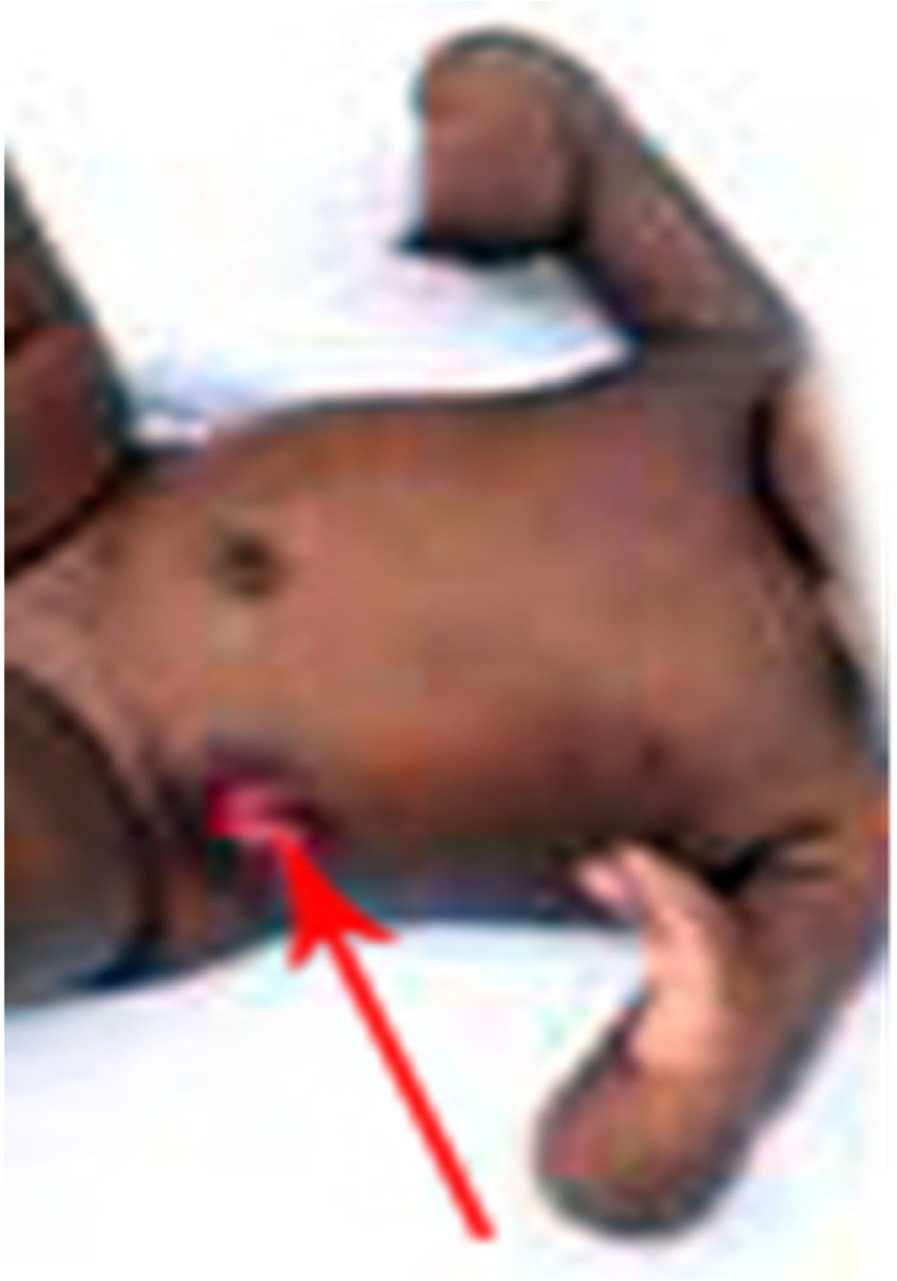
Bilateral radial club-hands and sigmoid colostomy. *Red arrow* points to “Sigmoid colostomy”

**Fig. 3:**
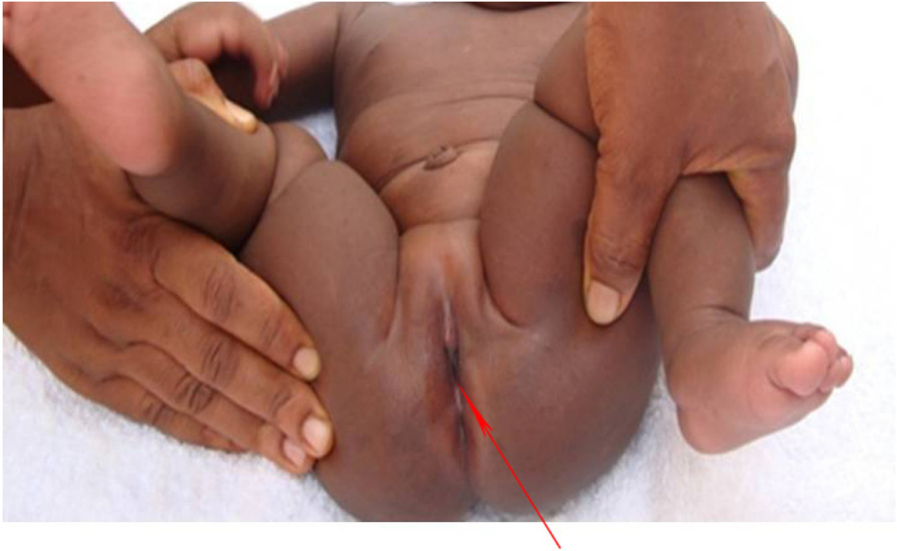
Absent anus and recto-vestibular fistula. *Red arrow* points to “Fistulous opening at the vestibule”

**Fig. 4:**
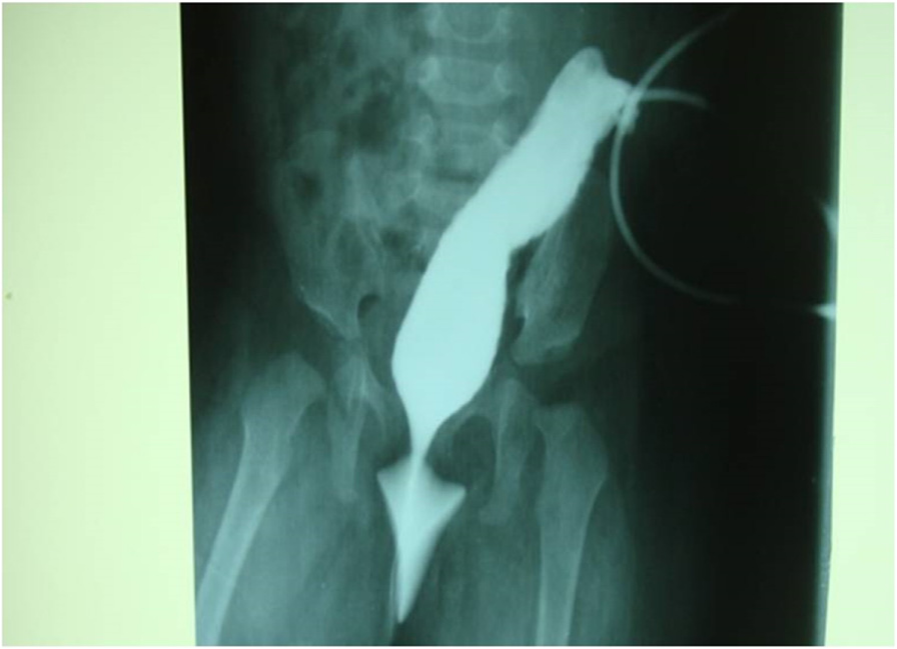
Colostogram demonstrating fistulous connection

**Fig. 5:**
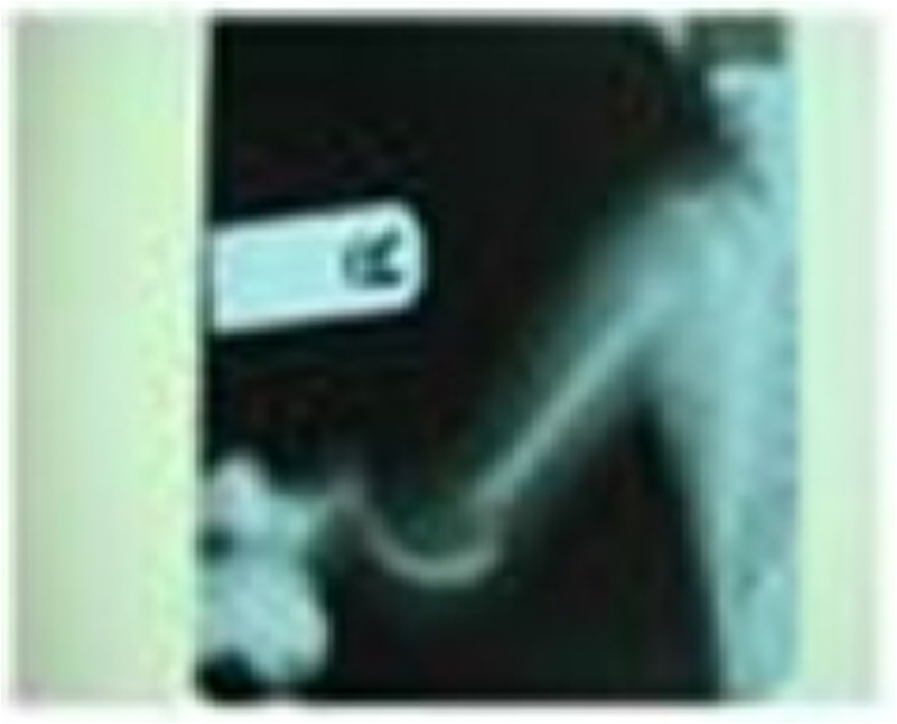
Plain X-ray of the right upper limb

**Fig. 6:**
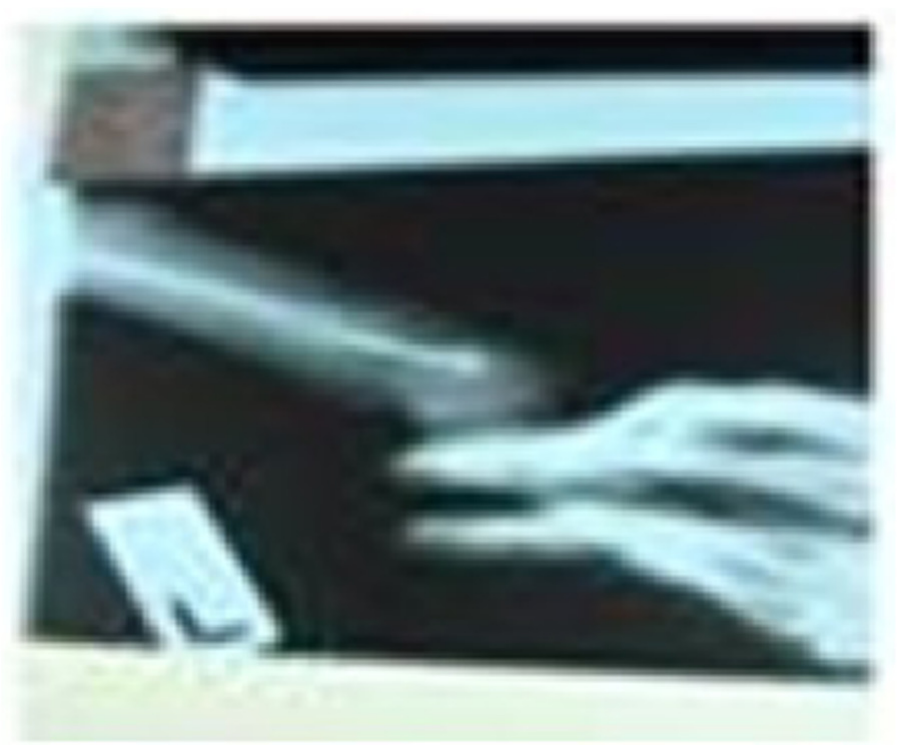
Plain X-ray of the left upper limb

Our patient had bilateral microphthalmia with bilateral corneal opacity (Fig. 1) and low-set ears. She had bilateral radial club-hands with radial deviation of both forearms as well as congenital absence of both thumbs (Fig. 2). Her anus was absent and a fistulous tract opened at the infantile vulval vestibule (Fig. 3). A gastrografin colostogram demonstrated a fistulous connection between the blind rectal pouch and the vestibule (Fig. 4). Plain X-rays of both her upper limbs revealed bilateral absence of the radial bones and deformed, shortened ulna bones as well as bony ankyloses of the elbow (Figs 5 and 6). Results from a platelet and full blood count were normal.

**Fig. 7:**
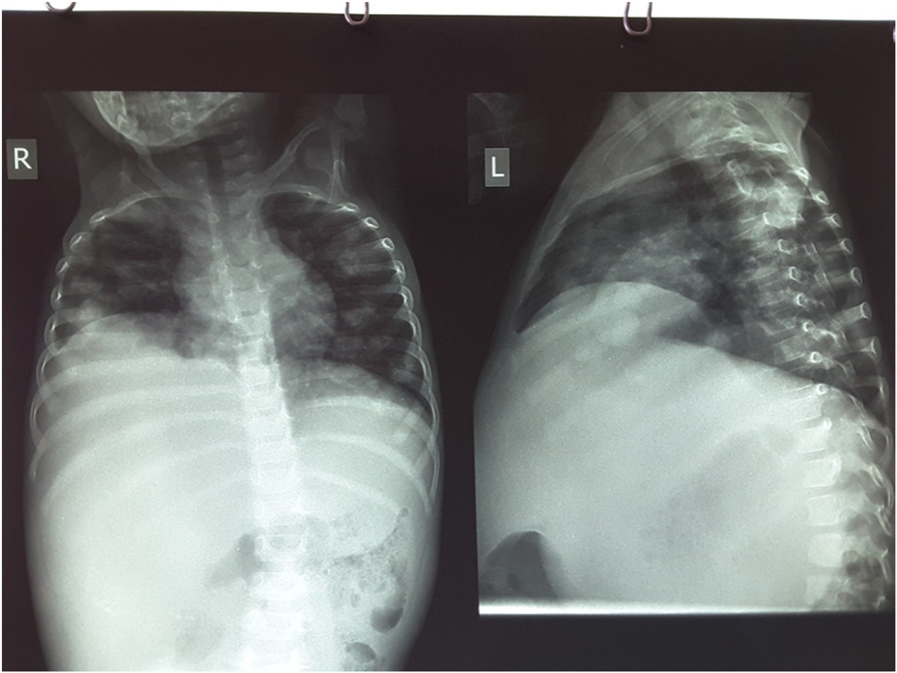
Plain chest X-ray showing cannonball metastases

She had a defunctioning sigmoid colostomy at 2 months followed by a posterior sagittal anorectoplasty after appreciable weight gain at 7 months and subsequent closure of the colostomy. A plan for corrective surgeries for other associated anomalies was being made when she developed hematuria and a right nephroblastoma with lung metastases (Fig. 7). As a result, further treatment was declined and our patient died from cardiopulmonary failure due to metastatic nephroblastoma.

## Discussion

Malformation syndromes associated with ARM are very rare. In a retrospective review of 103 consecutive patients with ARMs and their associated anomalies over a 22-year period, Cho *et al*. [2] observed malformation syndromes in only three patients. These consisted of cat eye syndrome, Opitz syndrome, and Potter syndrome type 1. By contrast, after a retrospective investigation of 317 children with ARMs from 1968 to 2001, Berger *et al*. [5] found associated syndromes in 26 children. The observed syndromes included Down syndrome (trisomy 21), OEIS (omphalocele-exstrophy-imperforate anus-spinal defects) complex, Currarino’s syndrome (sacral dysgenesis or agenesis, presacral tumor, and ARM), cat eye syndrome (numeric aberration of chromosome 22, pre-auricular fistula or auricular appendix, and cardiac malformation), radial aplasia x-linked syndrome (esophageal atresia type IIIb and bilateral radial aplasia), Johanson–Blizzard syndrome, Casamassima–Morton–Nance syndrome, and ROCA-Wiedemann syndrome (retardation of growth and development, ocular ptosis, cardiac defect, and anal atresia) [5]. In all they identified 18 syndromes in 26 children with ARMs. A VACTERL (vertebral, anorectal, cardiac, tracheal, esophageal, renal, and limb) association of anomalies commonly occurs with ARM. A VACTERL association is said to exist when three or more of these anomalies occur together with ARM [2, 3, 5].

Nevertheless, none of these reports found an ARM occurring in association with HOS. To the best of our knowledge, and based on a limited MEDLINE search, the occurrence of ARMs in association with HOS has not been previously reported, though more than 200 cases of the syndrome have been published in the literature [6].

HOS is an autosomal dominant disorder characterized by congenital cardiac and forelimb anomalies with a prevalence of 0.95 per 100,000 live births [7]. The underlying genetic defect is located on the long arm of chromosome 12 (12q^2^). Mutations in the *TBX3* and *TBX5* genes give rise to a wide range of phenotypes typical of HOS. These genes play a vital role in cardiac and skeletal development [7]. Mutations in these two T-box genes on chromosome 12q2 give an embryologic basis for the prevalence of atrial septal defects and VSDs in patients with HOS [8].

There is wide variability in the expression of both the cardiac and upper limb anomalies. While all patients with HOS have upper limb malformations, 85–95 % have cardiac anomalies [6]. Skeletal abnormalities mainly affect the upper limbs bilaterally, the radial ray predominantly, and the thumb particularly [6]. Thumb defects may be triphalangeal, hypoplastic, or complete absence [6]. Newbury-Ecob *et al*. [9] found cases in which the thumbs were normal. In our index case, both thumbs were completely absent. Poznanski *et al*. [10] demonstrated that carpal abnormalities were more specific for HOS than are changes in the thumb. Unfortunately, the radiographs of the upper extremities of our patient did not capture the skeletal details of her hand and we have lost the opportunity of repeating the radiographic examination because of her death. Skeletal involvement of the long bones of the upper limbs is also often bilateral and varied. It includes hypoplasia or complete absence of the radius as well as hypoplasia of the ulnar or even the humerus or both [7, 9, 10]. In our index case, there was complete absence of both her radial bones with deformity of her ulnar bones.

Almost every type of cardiac anomaly has been reported in HOS [11, 12]. However, secundum-type atrial septal defects (ASD) and VSDs are the most common [11, 12]. Our index patient had a pansystolic murmur that, on evaluation with echocardiography, was diagnosed as a VSD. Very rarely, some patients with HOS present with ocular defects [9], similar to the bilateral microphthalmia and bilateral corneal opacity in our patient. In addition, our patient also had microcephaly.

A diagnosis of HOS, therefore, usually requires the presence of cardiac malformations, conduction defects, and/or radial ray abnormalities in an individual, or the presence of radial ray abnormalities with or without cardiac malformation or conduction defects in those with a family history of HOS [13]. The fact that our patient’s parents, close relatives, and siblings who died from unrelated causes did not have abnormalities of the upper limbs and heart possibly makes this a case of sporadic rather than familial HOS.

The relationship between ARM and HOS is unknown. However, it has been observed that organs that originate from mesodermal tissues are often subject to malformations in children with ARM because of the embryopathology [5]. Similarly, the relationship between HOS and nephroblastoma is poorly understood and is a subject for further investigation. This relationship may be related to the mutations on the T-box genes that give rise to the embryologic defects in HOS.

Similar to most congenital anomalies, HOS can be diagnosed prenatally using three-dimensional sonography to depict the characteristic upper limb malformations. The radius and ulna can be seen easily at 13–16 weeks, while most cardiac anomalies except ASD and small VSD are clearly visible on US screening for anomalies at 18–20 weeks [14]. Therefore, a detailed sonography will reveal an abnormal four-chamber view of the heart and abnormalities of both hands and forearms that are consistent with bilateral radial agenesis [15]. Unfortunately, although US scans were performed at 5 and 8 months’ gestation, the anomalies in our index patient were not diagnosed.

## Conclusions

We report here the occurrence of ARM in association with HOS, an association that hitherto has not been reported. To enhance the prognosis and quality of life of children with syndromic ARM, prenatal US monitoring of high-risk pregnancies and expertise in prenatal detection of congenital anomalies are invaluable in ANC.

## Abbreviations

ANC, antenatal care; ARM, anorectal malformations; ASD, atrial septal defect; HOS, Holt–Oram syndrome; OEIS, omphalocele-exstrophy-imperforate anus-spinal defect; ROCA, retardation of growth and development, ocular ptosis, cardiac defect, and anal atresia; US, ultrasound; VACTERL, vertebral, anorectal, cardiac, tracheal, esophageal, renal, and limb; VSD, ventricular septal defect
